# Comparison on the Growth Variability of *Vibrio parahaemolyticus* Coupled With Strain Sources and Genotypes Analyses in Simulated Gastric Digestion Fluids

**DOI:** 10.3389/fmicb.2020.00212

**Published:** 2020-03-03

**Authors:** Yangmei Wang, Yong Zhao, Yingjie Pan, Haiquan Liu

**Affiliations:** ^1^College of Food Science and Technology, Shanghai Ocean University, Shanghai, China; ^2^Shanghai Engineering Research Center of Aquatic Product Processing & Preservation, Shanghai, China; ^3^Laboratory of Quality & Safety Risk Assessment for Aquatic Product on Storage and Preservation (Shanghai), Ministry of Agriculture Shanghai, Shanghai, China; ^4^Engineering Research Center of Food Thermal-Processing Technology, Shanghai Ocean University, Shanghai, China

**Keywords:** *Vibrio parahaemolyticus*, growth heterogeneity, simulate gastric fluids, maximum growth rate, gene heterogeneity

## Abstract

*Vibrio parahaemolyticus* is a food-borne pathogen that causes pathogenic symptoms such as diarrhea and abdominal pain. Currently no studies have shown that either pathogenic and non-pathogenic *V. parahaemolyticus* possess growth heterogeneity in a human environment, such as in gastric and intestinal fluids. The *tlh* gene is present in both pathogenic and non-pathogenic *V. parahaemolyticus* strains, while the *tdh* and *trh* genes are only present in pathogenic strains. This study firstly applied simulated human gastric fluids to explore growth variability of 50 strains of *V. parahaemolyticus* at 37°C. The bacterial growth curves were fitted by primary modified Gompertz model, and the maximum growth rate (μ*_*max*_*), lag time (LT), and their CV values were calculated to compare the stress response of pathogenic and non-pathogenic *V. parahaemolyticus* to simulated human gastric fluids. Results showed that the simulated human gastric fluids treatment significantly increased the μ_max_ of pathogenic strains and shortened the lag time, while decreased the μ_max_ of non-pathogenic strains and prolonged the lag time. Meanwhile, the CV values of genotypes (*tlh*^+^/*tdh*^+^/*trh*^–^) evidently increased, showing that the pathogenic genotype (*tlh*^+^/*tdh*^+^/*trh*^–^) strains had strong activity to simulated gastric fluids. All of the results indicated that the *V. parahaemolyticus* strains exhibited a great stress-resistant variability and growth heterogeneity to the simulated gastric fluids, which provides a novel insight to unlock the efficient control of pathogenic *V. parahaemolyticus*.

## Introduction

*Vibrio parahaemolyticus* is a gram-negative non-spore halophilic bacteria, which inhabits primarily coastal marine and estuarine environments and is widely found in various marine products (Alonsohernando et al., 2013). Cross-contamination due to the transportation and sale of seafood can also be found in large quantities in freshwater products (Jing et al., 2014). *V. parahaemolyticus* is a common food-borne pathogen, which can cause pathogenic symptoms such as diarrhea, vomiting, acute gastroenteritis, dehydration, shock, and even death after the ingestion of the bacteria (Baker-Austin et al., 2010). *V. parahaemolyticus* has become the major pathogen causing food-borne infection in many countries and coastal areas, such as Japan, Southeast Asia, the United States, and Taiwan (Yamasaki et al., 2009). According to reports of patients in China, due to *V. parahaemolyticus*, there have been 6.65 million cases of acute diarrhea, about 7.281 million cases of acute gastroenteritis, and the proportion of food-borne *V. parahaemolyticus* infection was about 68.0%. Thus, it is essential to control and prevent *V. parahaemolyticus*. Additionally, approximately 95% of *V. parahaemoliticus* cases are non-pathogenic and 5% are pathogenic in the environment (Nordstrom et al., 2007). The use of multiplex-PCR can comprehensively detect *V. parahaemolyticus*. All *V. parahaemolyticus* can amplify the *tlh* gene, which is unique to *V. parahaemolyticus*. The detection of *tdh* and *trh* genes as pathogenic isolates is also the main cause of human food-borne diseases (Letchumanan et al., 2015).

Although most environmental strains are not pathogenic, there are a small number of pathogenic strains with virulence factors that serve as the most critical in causing food-borne diseases. The reason that pathogenic *V. parahaemolyticuscan* causes human diarrhea, which is contained in seafood. And it is related to pathogenic factors, including the initial adhesion of pathogenic bacteria to host cells; and the release of pathogenic bacteria to the host cells with various biological activities such as toxins, proteins, and polysaccharides plays a toxic role. Usually, most pathogenic isolates and a few environmental sources of *V. parahaemolyticus* are pathogenic, which is associated with its cell-associated factors (such as colistin) (Obaidat et al., 2017), extracellular factors (such as hemolytic toxins) (Takahashi et al., 2000), and proteases (such as mucinase) (Jun et al., 2003).

Previous studies’ identified methods for the pathogenic isolates of *V. parahaemolyticus* mainly include: MLSA reveals that pathogenic isolates have a higher genetic diversity than non-pathogenic isolates (de Jesus Hernandez-Diaz et al., 2015). The pathogenic isolates include two hemolysin genes (*tdh* and *trh*) and a horizontally acquired type-three secretion system (T3SS2) (Xu et al., 2015). The comparative genomics method is used to analyze the differences in the genomes of pathogenic and non-pathogenic isolates, and there are more conserved genes in pathogenic isolates (Hazen et al., 2015). Using Whole Gene Sequencing (WGS) characterization, the pathogenic isolates containing *tdh* and *trh* genes account for the highest proportion of increasing cases of *V. parahaemolyticus* each year (Haendiges et al., 2015).

Current researches on *V. parahaemolyticus* have been mostly applied to single strain in pure cultures or in food. Studies have demonstrated that there are differences in the growth kinetics between temperature and salinity leading to pathogenicity and growth rate of the strains. There was an inseparable relationship between environmental factors and strain activity (Fujikawa et al., 2009). *V. parahaemolyticus* had different growth activities under four different temperature conditions (10, 20, 30, and 37°C) (Liu et al., 2016), and found that of the strains at 37°C, 3% had a good growth rate: As the temperature and salinity conditions become increasingly tense, their growth vigor was also more inhibited accordingly. Moreover, only a small amount of research has shown the pathogenicity of *V. parahaemolyticus*, which was present in the small intestine (Ritchie et al., 2012b), but few studies have been done on the growth kinetic parameters of *V. parahaemolyticus* under gastric digestion fluids. And there is also limited knowledge of the pathogenesis of diarrhea caused by *V. parahaemolyticus*. Therefore, it is of great significance to study the heterogeneity of growth of *V. parahaemolyticus* in gastric digestion fluids.

The main virulence factor of *V. parahaemolyticus* is hemolytic toxin—the others are heat-resistant hemolytic toxin (TDH), hemolytic toxin associated with heat-resistant hemolytic toxin (TRH), and heat-labile hemolytic toxin (TLH) (Takahashi et al., 2000; Dou et al., 2013). TDH is an enzyme that can digest the cell walls of blood cells and can produce a β-type hemolytic ring on blood agar medium, called Kanagawa (KP +) (Zhang and Chen, 2018). Almost all pathogens isolated *V. parahaemolyticus* produce a biologically active protein: TDH or TRH. The most direct characterization of the TDH-producing strain is the presence of beta-type hemolysis on the blood-stained plate, which is positive for Kanagawa (KP+) and is used as the most important marker for the pathogenicity of the strain. Pathogenic strains that produce only TRH (*trh*^+^), although negative for KP, also have enterotoxin activity, so these strains are generally considered to be pathogenic strains. Most environmental strains have neither *tdh* nor *trh*, and are called non-pathogenic strains. And epidemiological investigations have shown that the pathogenicity of *V. parahaemolyticus* is highly correlated with TDH (Guo et al., 2014). Otherwise, as a food-borne pathogen that is orally ingested, *V. parahaemolyticus* must survive human digestion to cause disease in humans. It is well known that in the gastric digestion fluids with pH 0.9–1.5, *V. parahaemolyticus* can neither survive nor cause people to become sick. However, the pH value of the stomach will rise rapidly within half an hour after ingestion. Therefore, for the food-borne pathogen *V. parahaemolyticus*, the bacterial concentration reaches 10^4^ CFU/ml, while the stomach provides favorable conditions for growth and colonization. It can cause other symptoms, such as diarrhea (Ritchie et al., 2012a).

Maximum growth rate (μ_max_) and lag time (LT) are the most important parameters for fitting microbiology models. Predicting microbes is a precise method for risk assessment (Lou et al., 2015). Currently, risk assessment studies based on *V. parahaemolyticus* focus on models established by single strains under different environmental conditions (Yang and Jiao, 2008). Few papers have analyzed the differences in the growth kinetic parameters of highly pathogenic *V. parahaemolyticus* from diverse sources. As *V. parahaemolyticus* is the most common pathogenic microorganism in aquatic products, establishing a perfect predictive model has market significance for ensuring the quality of aquatic products and predicting the shelf life of aquatic products.

## Materials and Methods

### Bacterial Strains and Preparation of Inoculum

This study was carried out in accordance with the recommendations of the World Medical Association’s Declaration of Helsinki and the Shanghai First Maternity and Infant Hospital Ethics Committee. The protocol was approved by the Shanghai First Maternity and Infant Hospital (ethics approval acceptance number: KS1940). The medical ethics committee approved the project to amend the project according to ethical requirements, conformed to ethical requirements, and agreed to implement it. All subjects gave written informed consent, or written informed consent was provided by parents/guardians for participants that were under the age of 16.

All *V. parahaemolyticus* virulence genes information were identified in our previous studies (Li et al., 2017; Niu et al., 2018a). A total of 50 strains of *V. parahaemolyticus* were used in this study, of which clinical isolates (VPC, *n* = 23) were recovered from the patients who presented with acute diarrhea in gastroenteritis outpatient clinics in the Shanghai hospital. And each fecal specimen was collected after informed consent was obtained from the patient or, if the patient was a child, from the child’s parent/guardian. Environment isolates (VPE, *n* = 27) were recovered from shrimp in freshwater or seawater in our previous study. The sources and genotypes of all of the strains are listed in Table 1. All *V. parahaemolyticus* strains were stored −80°C in 25% glycerol test tubes. Strains were streaked onto a thiosulfate-bile salt-sucrose agar medium (TCBS; Beijing Guotuqiao Technology Co., Ltd., Beijing, China) and cultured at 37°C for 8–12 h. A single colony from TCBS plate was transferred into 9 ml TSB (Beijing Luqiao Technology Co., Ltd., Beijing, China) at pH 8.0 and 3.0% (w/w) NaCl concentration, and then incubated overnight at 37°C and 220 rpm to prepare the test inoculum. The overnight culture was then diluted with 0.1% peptone water (PW; Beijing Luqiao Technology Co., Ltd., Beijing, China) to an optical density (OD) value for 1.2 ± 0.02 (about 10^9^ CFU/ml) at 600 nm (OD 600). The automated turbidimetric system Bioscreen C (Oy Growth Curves Ab Ltd., Raisio, Finland) was used to test the corresponding OD values at regular intervals (Niu et al., 2018b).

**TABLE 1:** The sources and genotypes of 50 strains of *V. parahaemolyticus.*

**No**	**Genotype**			**Source**	**No**	**Genotype**			**Source**
	***tlh***	***tdh***	***trh***			***tlh***	***tdh***	***trh***	
VPE01	+	–	+	Freshwater	VPE48	+	–	–	Freshwater
VPE02	+	–	–	Seawater	VPE49	+	–	–	Seawater
VPE03	+	–	+	Freshwater	VPC16	+	+	–	Pathogenic
VPE04	+	–	–	Freshwater	VPC18	+	+	–	Pathogenic
VPE05	+	–	–	Seawater	VPC25	+	+	–	Pathogenic
VPE07	+	+	–	Seawater	VPC26	+	+	–	Pathogenic
VPE08	+	–	+	Freshwater	VPC29	+	+	–	Pathogenic
VPE09	+	+	–	Seawater	VPC32	+	+	–	Pathogenic
VPE10	+	+	–	Seawater	VPC36	+	+	–	Pathogenic
VPE11	+	–	–	Seawater	VPC40	+	+	–	Pathogenic
VPE17	+	+	–	Seawater	VPC41	+	+	–	Pathogenic
VPE27	+	–	–	Freshwater	VPC44	+	+	–	Pathogenic
VPE28	+	+	–	Freshwater	VPC45	+	+	–	Pathogenic
VPE29	+	–	+	Freshwater	VPC46	+	+	–	Pathogenic
VPE32	+	–	–	Seawater	VPC47	+	+	–	Pathogenic
VPE36	+	–	+	Freshwater	VPC49	+	+	–	Pathogenic
VPE37	+	+	–	Seawater	VPC50	+	+	–	Pathogenic
VPE38	+	–	–	Freshwater	VPC51	+	+	–	Pathogenic
VPE40	+	–	+	Freshwater	VPC54	+	+	+	Pathogenic
VPE42	+	+	+	Human	VPC55	+	+	–	Pathogenic
VPE43	+	+	–	Human	VPC89	+	+	–	Pathogenic
VPE44	+	–	–	Freshwater	VPC90	+	+	–	Pathogenic
VPE45	+	–	–	Seawater	VPC94	+	–	+	Pathogenic
VPE46	+	–	–	Freshwater	VPC97	+	+	–	Pathogenic
VPE47	+	–	–	Freshwater	VPC100	+	+	–	Pathogenic

### Preparation of Simulated Digestion Fluids

Simulated human gastric fluids were prepared according to the formula of Minekus simulated digestive fluids (Minekus et al., 2014), and sterilized by 0.22 um filter. 1 M HCl and 1 M NaOH were used to adjust the pH (SGF was 4.0). The gastric fluids should be pre-warmed to 37°C before use, and to avoid precipitation, the CaCl_2_(H_2_O)_2_ solutions were finally added to the mixture. The above inoculum was appropriately serially diluted ten-fold with a 0.1% PW solution, mixed with SGF digestion solution, and treated at 37°C, 110 rpm constant temperature shaker for 120 min.

### Growth Curve Experiments

Microbial culture growth is often divided into four periods: lag time, logarithmic growth phase, stationary phase, and decay phase. The growth curve has the culture time as the abscissa and the logarithm of the number of bacteria or the growth rate as the ordinate. It represents the dynamic change of the whole process of growth, reproduction, and even death of bacteria in a new suitable environment. The automated turbidity system Bioscreen C (Oy Growth Curves Ab Ltd., Raisio, Finland) is used to detect the OD value of the wideband filter (600 nm) at regular time intervals. Changes in OD value can be observed throughout the total time period. The initial inoculum of each strain prepared was diluted ten-fold with five gradients in TSB. With strain concentration of approximately 10^4^ CFU/ml, the inoculated TSB was transferred to a 100-well microtiter plate and then placed in automated turbidimetric system Bioscreen C. Three OD measurement replicates were tested throughout this process. In addition, three independent experiments were performed at each growth condition, and there were three samples per strain completely utilized for testing. In this way, the total OD growth curve described will reach 900 patterns (3 replicates × 3 independent experiments × 2 growth conditions × 50 types of *V. parahaemolyticus*).

### Maximum Specific Growth Rate and Lag Time

Following the study by Lianou (Lindqvist, 2006; Alexandra and Koutsoumanis, 2011), the Bioscreen gradient dilution method is used to calculate the maximum specific growth rate of the strain as: log(Ni) = *K* − μ_max_ × t_*det*_.

In the formula: *t*_det_ is the time (h) when the OD 600 nm of *V. parahaemolyticus* reaches the Bioscreen C detectable level (10^6^–10^7^CFU/mL); μ_max_ is the maximum specific growth rate of *V. parahaemolyticus* growth rate [log CFU/(mL h)]; Ni is the initial concentration of *V. parahaemolyticus* (CFU/mL) in the selected sample well; and K is a constant.

Growth Kinetics Fitting: Using the modified Gompertz model, the growth data of *V. parahaemolyticus* under different growth conditions were fitted by Origin Pro 8.0 software (Origin Lab Corp., Northampton, MA, United States).

$$y=A+C\,\exp\,\left\{-\text{exp}\,\left[\frac{\mu_{\text{m}}}{A}\,\left(\lambda -t\right)+1\right]\right\}$$

were *A* means the initial total number of bacteria (log CFU/mL), *C* represents the difference between the maximum bacterial species and the initial bacterial species, log CFU/mL, μ_*m*_ represents maximum specific growth rate, λ is the time to reach the relative maximum growth rate (h), and y represents total number of bacteria at time (log CFU/mL).

### Statistical Analysis Methods

The prediction model is evaluated through the coefficient of determination *R*^2^, the root mean square error (RMSE), the Fisher’s *F* test *P*-value, the accuracy factor *A*_*f*_, the bias factor *B*_*f*_, and other parameters (Zhang et al., 2015). When *R*^2^ is close to 1, it means that the reference value of its prediction model is higher; otherwise, when it is close to 0, its reference value is lower. When *A*_*f*_ is equal to 1, it means that the predicted value and the observed value are equal. If the *A*_*f*_ value is larger, it indicates that the average accuracy of the model is lower. The *B*_*f*_ value in the range of (0.9–1.05) indicates that the prediction model has a small deviation. The parameter formula of each prediction model is as follows:

$$R^{2}=\left[1-\frac{\sum {\left(\text{pred}-\text{obs}\right)}^{2}}{\sum {\left(\text{obs}-\text{mean}\right)}^{2}}\right]$$

$$\mathrm{RMSE}=\sqrt{\frac{\sum {\left(\mathrm{obs}-\mathrm{pred}\right)}^{2}}{n}}$$

$$A_{\text{f}}=10\,\left(\frac{\sum \left|{\text{L}}_{\text{g}}\,\left(\mathrm{pred}/\mathrm{obs}\right)\right|}{n}\right)$$

$$B_{\text{f}}=10\,\left(\frac{\sum {\text{L}}_{\text{g}}\,\left(\mathrm{pred}/\mathrm{obs}\right)}{n}\right)$$

Under the condition of gastric digestive fluids treatment, the coefficient of variation (CV) of the μ_max_ is calculated as follows:

$$\mathrm{CV}=\frac{\mathrm{Standard}\,\mathrm{deviation}\,\mathrm{of}\,\mu_{\text{max}}}{\mathrm{Mean}\,\mathrm{value}\,\mathrm{of}\,\mu_{\text{max}}}\times 100\%$$

In addition, a significant difference test using *P*-values was also used to verify differences in growth rates of strains from different conditions. The level of statistical significance (*P* < 0.05) was tested by the least significant difference (LSD) method. Statistical analysis were analyzed using the SPSS statistical package 17.0 (SPSS Inc., Chicago, IL, United States).

## Results

### Changes in Maximum Growth Rate After Gastric Digestion Fluids Treatment

The growth curves of 50 strains of *V. parahaemolyticus* were measured in an automated turbidity system Bioscreen C. The Modified Gompertz model was used to evaluate μ_max_(Nauta and Dufrenne, 1999; Boonyawantang et al., 2012). Almost all test values were fitted to the equations given above statistical formula. All RMSE values tended to 0, and *A*_*f*_ and *B*_*f*_ were close to 1. The results showed goodness-of-fit with the Modified Gompertz model.

**FIGURE 1:**
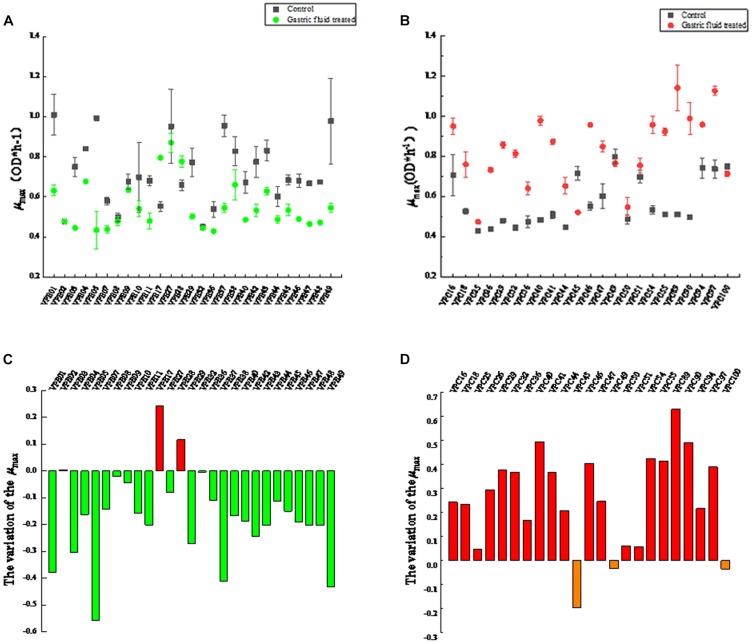
Maximum specific growth rates (μ_max_) of *V. parahaemolyticus* strains from different sources after SGF. **(A)** Maximum specific growth rate of environmental strains. **(B)** Maximum specific growth rate of pathogenic strains. **(C)** The change of maximum specific growth rate of environmental strains in SGF treatment. **(D)** The change of maximum specific growth rate of pathogenic strains in SGF treatment.

The maximum specific growth rates of *V. parahaemolyticus* from different sources after treatment with gastric digestive fluids (SGF) are shown in Figure 1. As shown in Figure 1A, which compares with control groups (before treatment with gastric digestive fluids), the μ_max_ of 89% of environmental *V. parahaemolyticus* strains were significantly reduced. Among them, the μ_max_ (OD × h^–1^) of VPE01, VPE05, VPE37, and VPE49 decreased sharply. The μ_max_ of VPE01 decreased from 1.001 to 0.63, the μ*_*max*_* of VPE05 went down from 0.889 to 0.434, the μ*_*max*_* of VPE37 dropped from 0.982 to 0.541, and the μ_max_ of VPE49 decreased from 0.979 to 0.565. Results indicated that the μ_max_ of environmental strains treated with gastric fluids were decreased, which had poor tolerance to gastric fluids and poor adaptability to external stress environments.

Interestingly, the μ_max_ changes of the pathogenic *V. parahaemolyticus* were completely opposite to that of environmental strains. The value of μ_max_ (OD × h^–1^) of most strains were significantly different (*P* < 0.01) between control and treatment groups. As shown in Figure 1B, the μ_max_ of most strains fluctuated between 0.4 and 0.6 in control groups. After gastric fluids treatment, the μ_max_ of the strains fluctuated between 0.64 and 1.1, increasing by 1.8 times. Among them, VPC40, VPC89, and VPC90 increased significantly. The μ_max_ of VPC40 increased from 0.483 to 0.979, the μ_max_ of VPC89 increased sharply from 0.511 to the highest point of 1.142, and the μ_max_ of VPC90 increased from 0.498 to 0.989. Also as shown in Figure 1D, the μ_max_ changes of VPC40, VPC89, and VPC90 were 0.496, 0.631, and 0.491, respectively. Other pathogenic isolates had increased to varying degrees. Meanwhile, the μ_max_ of VPC45, VPC49, and VPC100 decreased on the contrary: The reductions were 0.196, 0.013, and 0.036, respectively. The reason for these reductions will be the focus of future experiments.

### Tendency of Lag Time (LT) (λ)

**FIGURE 2:**
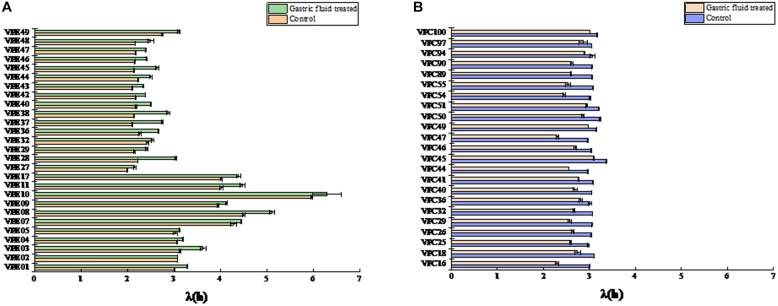
The lag time (λ) of *V. parahaemolyticus* strains from different sources after SGF. **(A)** Lag time (λ) of environmental strains. **(B)** Lag time (λ) of pathogenic strains.

The biologically accepted definition is the time when a microbial population undergoes a mutation in the environment and begins to reproduce after self-adjustment. In the geometric sense, the microbial lag period refers to the time interval when the logarithmic growth phase of the microorganism reaches the maximum specific growth rate (Aguirre and Koutsoumanis, 2016). The lag time (LT) exhibited changes, as shown in Figure 2. The LT of most strains were significantly different (*P* < 0.05) between control and treatment groups. In control groups, the LT values of environment-derived isolates were different, with about 60% of the isolates strains having LT of less than 3 h (Figure 2A). The LT of 92.5% of the strains increased in the treatment groups after SGF, on the other hand. Among them, the LT of VPE10 reached a maximum of 6.2965, the LT of VPE27 fell to a low of 2.1563, and the LT of VPE36 and VPE02 fluctuated insignificantly. However, as shown in Figure 2B, for pathogenic-derived strains, the LT of the strains was about 3 h, which was not significantly different in control groups. While, after SGF treatment, the LT of almost all pathogenic-derived isolates strains were shortened significantly, and about 91% of the strains had a LT of less than 3 h. The shortening of the pathogenic strains LT corresponded to the stress of gastric fluids treatment.

### Evaluation of Growth Variability After SGF

**FIGURE 3:**
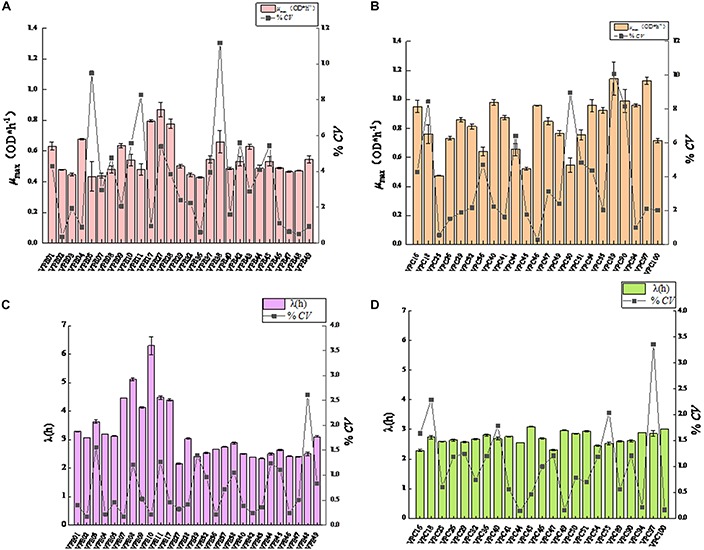
Mean value curve of maximum specific growth rates (μ_max_) and coefficient of variation curve of μ_max_ among strains (CV-Strain) after SGF treatment. **(A)** Mean value curve of maximum specific growth rates (μ_max_) and coefficient of variation curve of μ_max_ among strains (CV-Strain) of environmental strain. **(B)** Mean value curve of maximum specific growth rates (μ_max_) and coefficient of variation curve of μ_max_ among strains (CV-Strain) of pathogenic strain. **(C)** Mean value curve of lag time (λ) and coefficient of variation curve of lag time (λ) among strains (CV-Strain) of environmental strains. **(D)** Mean value curve of lag time (λ) and coefficient of variation curve of lag time (λ) among strains (CV-Strain) of pathogenic strains.

In order to study the effect of SGF treatment on the growth rate of *V. parahaemolyticus*, the growth variability of the strain was studied by coefficient of variation (CV). The maximum specific growth rate and CV values of environmental isolate strains after SGF treatment were shown in Figure 3A. After SGF treatment, the maximum specific growth rate of the environmental isolate strains decreased, and the CV value of the strain μ_max_ also fluctuated. When the μ_max_ of VPE1, VPE5, VPE37, and VPE49 decreased the most, the CV values of μ_max_ were 4.3%, 9.5%, 3.9%, and 0.9%, respectively. The results showed that the CV value of μ_max_ of VPE5 was the largest, which revealed the largest growth heterogeneity in the gastric fluids environment, and there was growth heterogeneity between the various strains of *V. parahaemolyticus* after SGF treatment.

Results depicted the difference in the μ_max_ coefficient of variation CV of pathogenic isolate strains after SGF treatment (Figure 3B). The CV value of most strains fluctuated between 0.26 and 4.00%. After SGF treatment, the CV value of μ_max_ of VPC18, VPC50, VPC89, and VPC90 increased sharply, which were 8.44, 8.97, 10.07, and 8.17%, respectively. The CV values of μ_max_ of VPC25 and VPC46 fell respectively by 0.55 and 0.26%. The CV values of μ_max_ of pathogenic isolates, which were easier to grow in gastric fluids, were significantly higher than those of environmental isolates. When pathogenic *V. parahaemolyticus* grows to a pathogenic amount in human stomach, it has the potential of causing diarrhea and gastroenteritis.

The CV value of LT was significantly different (*P* < 0.01) between environmental isolates and pathogenic isolates after SGF treatment (Figure 3C). As shown in Figure 3C, the CV values of LT of environmental isolates fluctuated between 0.16% and 1.50%, while the CV values of LT of VPE03 and VPE48 exceeded 1.5%, which were 1.56 and 2.61%, respectively. As shown in Figure 3D, the CV values of LT of pathogenic isolates fluctuated between 0.14 and 1.3%, while all of the CV values of LT of VPC16, VPC18, VPC40, VPC55, and VPC97 strains were larger than the fluctuation range, which were 1.64, 2.29, 1.78, 2.04, and 3.35, respectively. By comparing the growth kinetic parameter, the CV value of μ_max_ and LT more comprehensively represents the growth heterogeneity for *V. parahaemolyticus* strains.

### Comparison of Growth Variability From Different Sources

**FIGURE 4:**
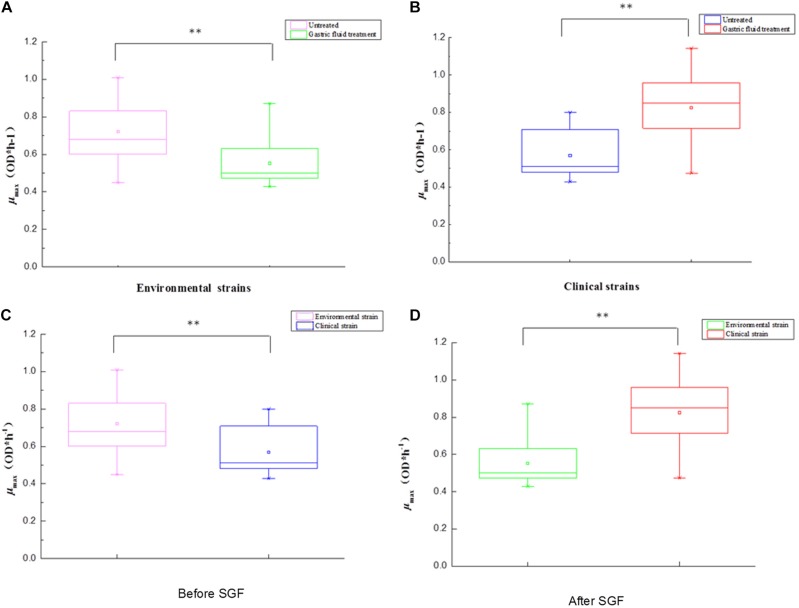
The box plot between environment and pathogens for *V. parahaemolyticus* strains under gastric fluids treatment in the following growth conditions. **(A)** The environmental strains with *P* = 0.001 (control group and SGF treatment). **(B)** The pathogenic strains with *P* = 0.000 (control group and SGF treatment). **(C)** The environmental strains and pathogenic strains with *P* = 0.001 before SGF treatment. **(D)** The environmental strains and pathogenic strains with *P* = 0.000 after SGF treatment. ^∗∗^Statistical significance (*p* < 0.05).

Table 1 shows the sources of different types of *V. parahaemolyticus*, divided into environmental and pathogenic strains. Among them, environmental strains can be roughly divided into two categories: freshwater and seawater. The box diagrams of the four types are: environment group, pathogenic group, different sources strains before SGF treatment, and different sources strains after SGF treatment (Figure 4). The average of the maximum specific growth rate μ_max_ was counted, and significant differences were calculated by the *P*-value. In addition, the *P*-values of the differences were 0.001 [environment group, SGF treatment (Figure 4A)], 0.000 [pathogen group, SGF treatment (Figure 4B)], 0.001 [environment group and pathogenic group before SGF (Figure 4C)], and 0.000 [Environment group and pathogenic group after SGF (Figure 4D)]. All four groups performed significant difference analysis.

### Effects of Genotypes on Growth Variation After Treatment With Different Sources

Genetic heterogeneity has a certain influence on the growth variation of *V. parahaemolyticus*. To further describe the growth characteristics of *V. parahaemolyticus*, 50 strains from different sources (shown in Table 1) were classified by genotype. Based on different virulence factors, *V. parahaemolyticus* could be divided into four categories: (1) *tlh^+^/tdh^–^/trh^–^*, (2) *tlh^+^/tdh^+^/trh^–^*, (3) *tlh^+^/tdh^–^/trh^+^*, and (4) *tlh^+^/tdh^+^/trh^+^* (Letchumanan et al., 2014), which were used to explore the internal causes of the growth variability of *V. parahaemolyticus*. In these virulence genes, all clinical and environmental strains of *V. parahaemolyticus* had been expressed as *tlh* in previous studies (Okada et al., 2009). Today, the most important virulence factors of the recognized *V. parahaemolyticus* are heat-resistant direct hemolysin (TDH) and heat-resistant hemolysin (TRH), which are encoded by the *tdh* and *trh* genes, respectively, as an important basis to distinguish pathogenic and non-pathogenic *V. parahaemolyticus.*

**FIGURE 5:**
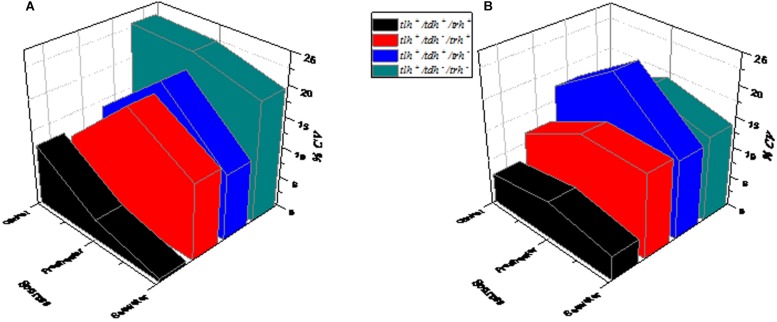
The influence of the genotype on the growth variability of *V. parahaemolyticus* strains from different sources. **(A)** Control group (not treated with SGF). **(B)** After SGF treatment.

The changes of *V. parahaemolyticus* of different genotypes after SGF treatment are shown in Figure 5. In control conditions, *tlh^+^/tdh^–^/trh^–^* (green) exhibited the maximum growth variability compared to the other three genotypes, which had CV values at higher levels (Figure 5A). As shown in Figure 5B, *tlh^+^/tdh^+^/trh^+^* (black) and *tlh^+^/tdh ^–^/trh^+^* (red) performed moderate variability in growth. After SGF treatment, the CV value of *tlh^+^/tdh^+^/trh ^–^* (blue) was the highest of those of other genotypes (*tlh^+^/tdh^–^/trh^–^*, *tlh^+^/tdh^–^/trh^+^*, and *tlh^+^/tdh^+^/trh^+^*).

## Discussion

### Effects of SGF on Growth Variability

Predictive microbiology model is an important research topic in the field of food safety (Yoon et al., 2008; Stan-Lotter et al., 2010). One should use a modified Gompertz model for growth kinetic analysis of pathogenic and non-pathogenic *V. parahaemolyticus* in broth and oysters, and use two-dimensional models (such as Davey and square root models) to fit lag time and the maximum growth rate (Yoon et al., 2014). Pathogenic *V. parahaemolyticus* strains in *Vannamei* have different growth kinetic parameters (Lou et al., 2015). The prediction model of *V. parahaemolyticus* was used to study the growth variation of pathogenic and non-pathogenic strains. The maximum specific growth rate (μ_max_) and lag time (LT) are the two most important parameters of microbial dynamics. It was obvious that 37°C was considered to be the optimal growth condition in the control group (Figure 1), which had already been verified by the similar completion in laboratory experiments (Baker-Austin et al., 2010; Fernandez-Piquer et al., 2011). However, the most effective obstacle of the survival and onset of *V. parahaemolyticus* is the human stomach. After SGF treatment, *V. parahaemolyticus* showed different variability. The maximum specific growth rate of environmental strains decreased (Figure 1A), while the lag time was increased simultaneously (Figure 2A). In fact, most environmental strains were adapted to the natural environment, while SGF was not adjusted to the growth of those strains. Interestingly, the growth of the pathogenic *V. parahaemolyticus* was opposite to SGF stress. The maximum specific growth rate of the pathogenic strains increased (Figure 1B), whereas the lag period was shortened (Figure 2B). The value of μ_max_ of pathogenic VPC89 was 1.1418, and the lag time was only 2.59 h. This data indicates that VPC89 had a short lag time, rapidly entered into the log phase, which grew rapidly to reach the maximum bacterial concentration in a short time. Studies have shown that the length of the lag time can reflect the strain’s ability to respond to the new environment. The CV value of μ_max_ and LT more clearly reflected the heterogeneity of *V. parahaemolyticus* after SGF (Figure 3). And the pathogenic bacteria was significantly different (*P* < 0.01) than that of environmental strains (Figure 4), which meant the pathogenic bacteria had a stronger ability to adapt to the SGF environment. This situation brought certain risks to food control and to human health (Arroyo-Lopez et al., 2014).

### Impact of SGF on Pathogenicity

In this study, the differences in maximum growth rates and lag time between pathogenic and non-pathogenic isolates were observed after SGF treatment. The *V. parahaemolyticus* with high maximum growth rates in SGF generally presented a positive correlation with virulence factors and pathogenicity. Extracellular enzymes, endotoxin lipopolysaccharides, and heat-labile capsular polysaccharides have enhanced the pathogenicity of pathogenic strains. The pathogenic *V. parahaemolyticus* contains the *tdh* gene (Petronella and Ronholm, 2018), which is located in the coding region adjacent to the chromosome. The *tdh* gene with genetic characteristics was more easily expressed than the *trh* gene during SGF (Yoshitsugu et al., 2013; Shimohata and Takahashi, 2010).

According to the strain information data collected in Table 1, since there was only one strain containing *tlh*^+^/*tdh*^+^/*trh*^+^, the characteristics of this genotype would not be typical, and the black curve provided some references for different trends. Besides, the genotype *tlh^+^/tdh^+^/trh*^–^ is found in almost all pathogenic isolated strains. In addition, the secretory systems of *V. parahaemolyticus* play an indispensable role in the pathogenesis. Almost all pathogenic *V. parahaemolyticus* have a type III secretion system (T3SS), which is the main contributor to the pathogenicity of *V. parahaemolyticus* (Rohinee et al., 2012). The type III secretion system is a bacterial protein secretion device, which mediates bacterial cytotoxicity in a manner that induces programmed cell death or autophagy of host cells (Kwon-Sam et al., 2004; Okada et al., 2010). Li et al. (2016) reported that the *vtr*A/*vtr*C complex of *V. parahaemolyticus* can induce *vtr*B to activate T3SS2, and *vtr*A and *vtr*B deletion mutants cannot produce TDH and T3SS2 related proteins (Gotoh et al., 2010). *vop*V, *vop*Z, and *vop*L are the three major secretory effector proteins of T3SS2, and they play an important role in the pathogenicity and intestinal colonization of hemolytic *V. parahaemolyticus*.

These facts indicated that the maximum growth rate of pathogenic strains was increased, the lag time was decreased after SGF treatment, and the CV values of genotypes (*tlh*^+^/*tdh*^+^/*trh*^–^) increased evidently more than those of other genotypes (*tlh^+^/tdh^–^/trh^–^*, *tlh^+^/tdh^–^/trh^+^* and *tlh^+^/tdh^+^/trh^+^*), as shown in Figure 5. Therefore, the results suggested a relationship between the expression of virulence genes and the (*tlh*^+^/*tdh*^+^/*trh*^–^) genotype of *V. parahaemolyticus*, and it was more adaptable to the changes of gastric fluids environment.

## Conclusion

The growth kinetics characteristics of 50 pathogenic and non-pathogenic *V. parahaemolyticus* strains with different genotypes and from different sources were determined in simulated gastric fluids. Compared to the control group, the μ_max_ of 87% of pathogenic strains significantly increased, while the μ_max_ of 89% of non-pathogenic strains decreased. However, the LT of all pathogenic strains was shortened, and the LT of all non-pathogenic strains was prolonged in simulated gastric fluids. Meanwhile, the CV values of genotypes (*tlh*^+^/*tdh*^+^/*trh*^–^) increased evidently more than those of other genotypes (*tlh^+^/tdh^–^/trh^–^*, *tlh^+^/tdh^–^/trh^+^*, and *tlh^+^/tdh^+^/trh^+^*). Therefore, more attention should be paid to the clinical genotypes (*tlh*^+^/*tdh*^+^/*trh*^–^) associated with severe virulence, which is of great significance for the prevention and maintenance of human health. The generated knowledge will facilitate the incorporation of strain variability in predictive microbiology and microbial risk assessment and will hence provide an efficient way and scientific guidance to control the pathogens in the food industry.

## Data Availability Statement

All datasets generated for this study are included in the article/supplementary material.

## Author Contributions

YZ, YP, and HL conceived and supervised the study. YW designed and performed the experiments, analyzed the data, and wrote the manuscript. YZ and HL revised the manuscript.

## Conflict of Interest

The authors declare that the research was conducted in the absence of any commercial or financial relationships that could be construed as a potential conflict of interest.
